# Technical Note: Determination of mercury in blood by on-line digestion with FIMS

**DOI:** 10.1155/S1463924696000260

**Published:** 1996

**Authors:** Tiezheng Guo, Jörn Baasner

**Affiliations:** Bodenseewerk Perkin-Elmer GmbH Überlingen Germany


Journal of Automatic Chemistry, Vol. 18, No. 6 (November–December 1996) pp. 217–220

### Technical Note: Determination of mercury in blood by on-line digestion with FIMS

**Tiezheng Guo and Jörn Baasner**

*Bodenseewerk Perkin-Elmer GmbH, Überlingen, Germany*

This technical note reports on an evaluation of an FIAS method [1, 2] using a dedicated mercury system, the Perkin-Elmer Flow Injection Mercury System (FIMS) with an AS-90 autosampler. A Prolabo Maxidigest MX-350 was used for on-line digestion of blood samples. The MX-350 was controlled by the FIMS using the recommended interface.

Figure 1 shows the complete manifold configuration used for this determination. A more detailed description of the manifold configuration for the generation of the mercury vapour is given in figure 2.

When installing the cooling system, care was taken to ensure that aspiration of the cooling water occurs at the bottom of the supply vessel, while intake is as close as possible to the top. This guaranteed that the cooling water had a low, even temperature at all times. Table 1 lists the tubing used.

The carrier gas flow rate was approximately 100 ml/min.

### Recommendations

A 1 m length of PTFE tubing was placed between the gas–liquid separator and the absorption cell to minimize any water vapour transferred to the cell. A plastic gas–liquid separator (B050-7959), equipped with a PTFE membrane filter, placed after the glass type separator was also shown to help reduce water vapour—see figure 3. The use of the second gas liquid separator slightly reduced the sensitivity of the mercury measurements.

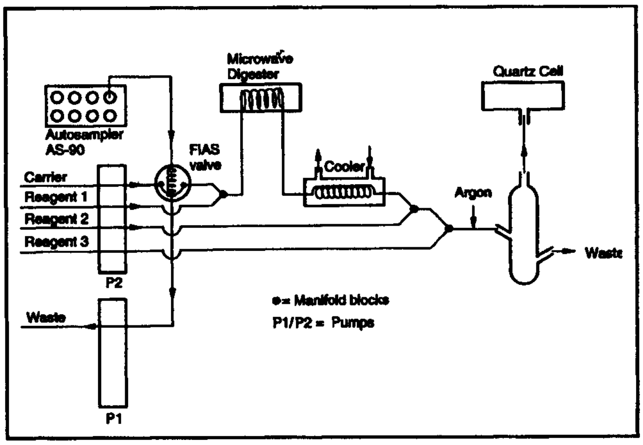

*Figure 1. FIMS with on-line microwave digestion.*

*Table 1. Pump tubing used.*

| Solution | Color code | I.D. (mm) | Flow rate (ml/min) |
|---|---|---|---|
| Pump 1: | | | |
| Sample | Yellow/blue | 1·52 | 9–11 |
| Cooling water | Black/white | 3·18 | 32–35 |
| Pump 2: | | | |
| Carrier solution | Yellow/blue | 1·52 | 9–11 |
| Acid (HCl) | Red/red | 1·14 | 5–6 |
| $KMnO_4$ | Red/red | 1·14 | 5–6 |
| $NaBH_4$ | Red/red | 1·14 | 5–6 |
| Waste | Violet/violet | 2·06 | 35–40 |

### Reagents and solutions

All reagents used were of analytical-reagent grade, unless otherwise stated.

*Reducing solution:* 0·05% (m/v) $NaBH_4$ in 0·05% (m/v) NaOH (0·5 g $NaBH_4$ + 2 NaOH pellets/l solution). 1·5 ml Dow Corning 110 A antifoaming agent was added to 1 l of the reduction solution. This solution was prepared every day.

*Carrier solution:* deionized water.

*Digestion solution:* 5% (v/v) HCl (50 ml 30% suprapur HCl in 1 l water).

*$KMnO_4$ solution:* 0·05% (m/v); 0·05 g of $KMnO_4$ was dissolved in 1 l of deionized water. Care was taken to ensure that the $KMnO_4$ crystals dissolved completely. The solution was stored in a dark bottle and was prepared weekly.

*Oxidation reagent:* 2 g KBr and 0·56 g $KBrO_3$ were dissolved in 25 ml deionized water. This solution was prepared weekly.

*Triton solution:* 1·2% (m/v) Triton X-100; 1·2 g of Triton X-100 was dissolved in 100 ml deionized water.

*Stabilization solution:* 0·5% (m/v) $K_2Cr_2O_7$; 0·5 g $K_2Cr_2O_7$ was dissolved in 100 ml of 1 + 1 (v/v) nitric acid.

*Hg stock solution I:* 1000 mg/l.

*Hg stock solution II:* 10 mg/l; Hg stock solution I was diluted 100× with 10% (v/v) HCl (concentrated HCl diluted 1:10). This solution was found to be stable for several months.

Reference solutions in the range 100–500 µg/l were prepared by further dilution of stock solution II. These reference working solutions must contain 1% (v/v) of the stabilization solution.

0142-0453/96 $12·00 © 1996 Taylor & Francis Ltd

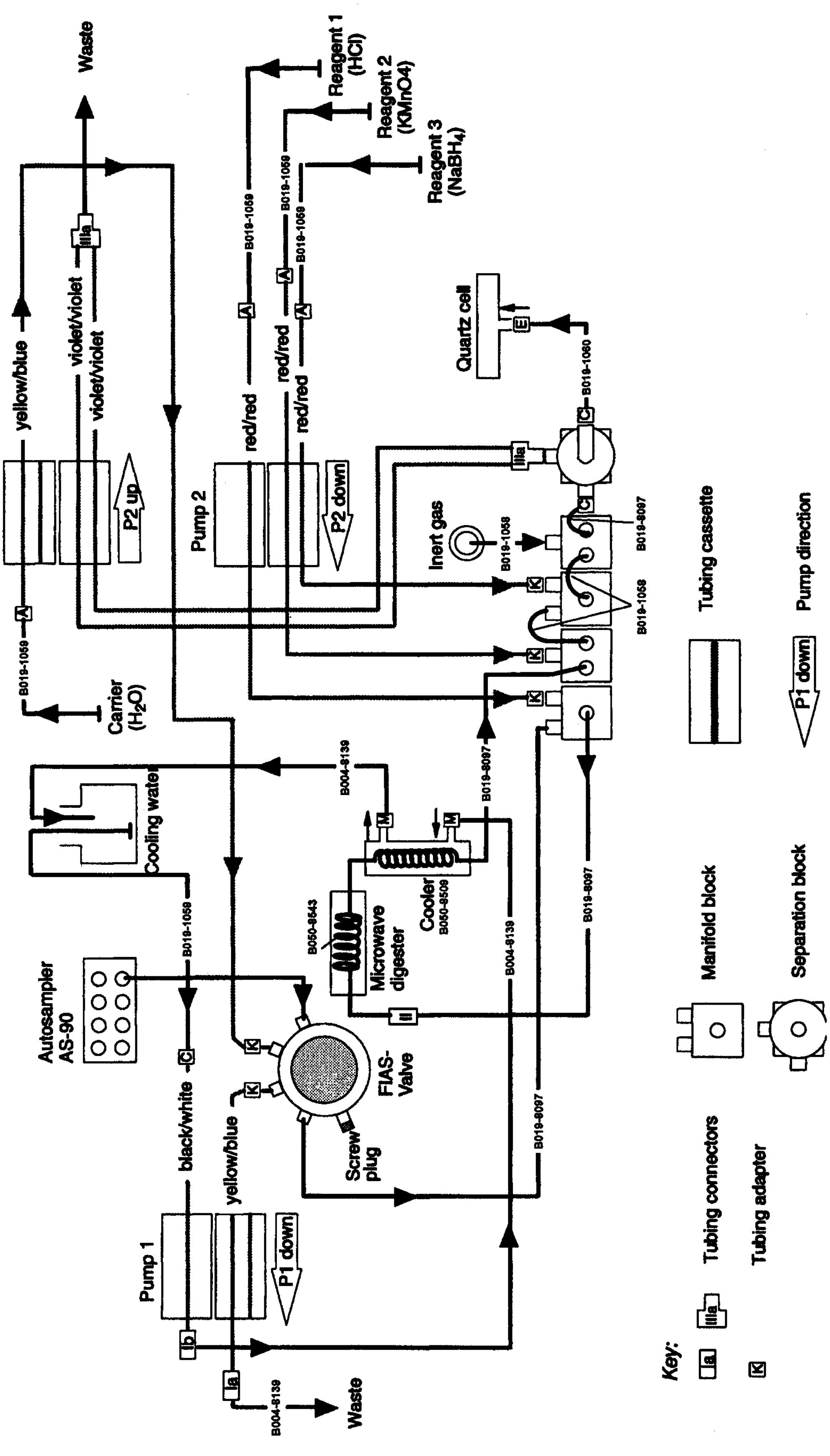

*Figure 2. Connection diagram for the FIMS with on-line microwave digestion.*

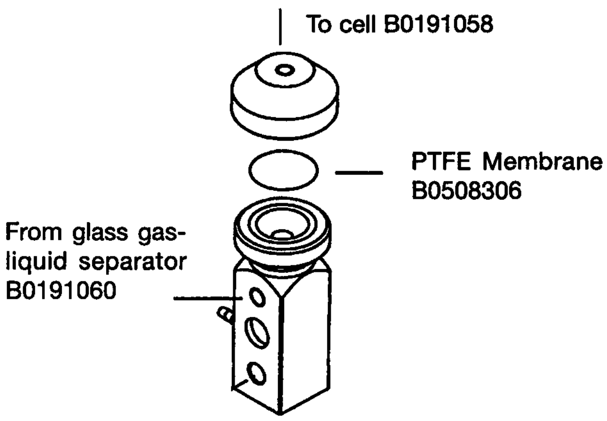

*Figure 3. Use of an additional gas–liquid separator to reduce water vapour.*

For standard addition calibration, appropriate amounts of the reference working solutions were added to one of the blood samples.

### Sample preparation

0·5 ml of Triton solution and 0·5 ml of blood sample were pipetted into a 10-ml polystyrene test tube and then diluted with water to 5 ml. 0·5 ml of oxidation reagent was added and the solutions mixed.

### *Preparation of the standard addition solutions*

The blank solution was prepared in the same way as the sample. However, only 0·2 ml of the oxidation reagent was added—larger quantities of the oxidation reagent may create a background absorption signal which affects the results.

Triton solution was added to each aliquot of the blood sample or standard and the mixture was diluted to approximately 4 ml with water. The appropriate volume of reference solution was added and diluted to 5 ml. Then 0·6 ml of oxidation reagent was added. This ensures that the acid contained in the reference solutions does not cause the samples to coagulate immediately.

Table 2 gives information on preparing a series of standard addition solutions covering a range of 5 to 20 μg/l Hg in blood. Table 3 also provides similar information for a concentration range of 10 to 100 μg/l Hg in blood.

When working at higher concentration levels, a higher concentration reference solution should be used to minimize added solution volume and avoid coagulation of the sample.

### Measurements

Before the first measurement is made, the FIAS pump 2 was started, pumping water instead of the $KMnO_4$ solution. $KMnO_4$ should be pumped only after HCl and $NaBH_4$ are available in the complete system. This ensures that no $MnO_2$ is formed as a reduction product of $KMnO_4$ and deposited in the tubing or the gas–liquid separator. Deposited $MnO_2$ markedly reduces the sensitivity of the Hg measurements.

*Table 2. Parameters for standard addition calibration in the range 5–20 μg/l Hg.*

| Solution | Volume [μl] of 100 μg/l-reference solution | Hg concentration in the test solution [μg/l] | Equivalent Hg concentration in undiluted blood [μg/l] |
|---|---|---|---|
| Blank | 0 | — | — |
| Sample | 0 | — | — |
| Add. 1 | 25 | 0·5 | 5 |
| Add. 2 | 50 | 1 | 10 |
| Add. 3 | 75 | 1·5 | 15 |
| Add. 4 | 100 | 2 | 20 |

*Table 3. Parameters for standard addition calibration in the range 10–100 μg/l Hg.*

| Solution | Volume [μl] of 100 μg/l-reference solution | Hg concentration in the test solution [μg/l] | Equivalent Hg concentration in undiluted blood [μg/l] |
|---|---|---|---|
| Blank | 0 | — | — |
| Sample | 0 | — | — |
| Add. 1 | 10 | 1 | 10 |
| Add. 2 | 20 | 2 | 20 |
| Add. 3 | 50 | 5 | 50 |
| Add. 4 | 100 | 10 | 100 |

As a precautionary measure, The tubing system was flushed after the analysis was completed. The system was first flushed by pumping 1 + 1 (v/v) $HNO_3$ followed by deionized water.

Since the microwave operation heats the entire system and Hg-vapour generation and the transfer of mercury to the gas phase are temperature-dependent, it was necessary to stabilize the temperature of the system prior to the actual measurements. This was achieved by running the FIAS program for 5–10 minutes, for example for six repeat measurement cycles. Then the measuring program was started. Reducing agent, acid, etc. were pumped during this temperature stabilization phase.

To test the procedure, a variety of organic Hg species were analysed. A recovery of 100 ± 2% was achieved for all species tested.

Comparison with an off-line digestion procedure and with a direct procedure using amalgamation confirmed the need to digest the blood samples in order to obtain accurate results. The comparison showed that on-line digestion was superior to the off-line method with regard to sample throughput, sample consumption, and total measurement time.

More information on this and on the system components used (3D digestion loop, cooler, etc.) is provided by Guo *et al.* [1].

*Table 4. Determination of Hg (μg/l) in blood control samples.*

| Control sample | Measured value | Nominal value |
|---|---|---|
| Seronorm No. 010012 | 3·9 ± 0·4 | 4·9 (4·1–5·2) |
| Seronorm No. 205052 | 3·1 ± 0·4 | 5* |
| Seronorm No. 203056 | 9·6 ± 0·9 | 10* |

* Provided by the manufacturer

The accuracy of the procedure was tested with control samples. Table 4 lists the results obtained. The linear range extends to approximately 300 μg/l in blood. The detection limit is 0·1 μg/l Hg in blood with a sample volume of 500 μl and dilution of the original sample by a factor of 10. Sample throughput is approximately 45 measurements per hour.

Due to the fact that the spectral bandpass of a FIMS System is relatively wide compared to a 'normal' AA spectrometer, special care had to be taken that hydride forming elements did not cause a non specific absorption. The most likely elements which could show such a behaviour are arsenic and bismuth. The reported concentrations of As in blood are in the range of 0·0042 to 0·19 mg/l and for Bi 0·002–0·023 mg/l [3]. Concentrations of up to 0·5 mg/l for As and 0·2 mg/l for Bi were added to blood samples before the on-line digestion procedure.

The additions did not affect the measurements of Hg in blood. So it can be concluded, that normal and even higher levels of hydride forming elements do not show any interference effect.

### Summary

The flow injection technique described, accurately determines total mercury content in blood. Only minimal sample preparation is necessary. All steps, such as microwave digestion and addition of further reagents, take place automatically.

### References

1. Guo, T. and Baasner, J., *Talanta*, **40** (1993), 12.

2. Guo, T. and Baasner, J., *Determination of Mercury in Blood by On-line Digestion with Flow Injection AAS* (Perkin-Elmer Corporation).

3. Ivengar, G. V., Kollmer, W. E. and Bowen, H. J. M., *The Elemental Composition of Human Tissues and Body Fluids: A Compilation of Values for Adults*. (Verlag Chemie GmbH, Weinheim, 1978).

